# An anti-leakage liquid metal thermal interface material

**DOI:** 10.1039/d0ra02351e

**Published:** 2020-05-18

**Authors:** Kaiyuan Huang, Wangkang Qiu, Meilian Ou, Xiaorui Liu, Zenan Liao, Sheng Chu

**Affiliations:** State Key Laboratory of Optoelectronic Materials and Technologies, Sun Yat-Sen University China chusheng@mail.sysu.edu.cn; Huwei Technologies Co., Ltd Shenzhen 518129 China; Guangtai Technology Co., Ltd Dongguan 523590 China

## Abstract

Liquid metals (LMs) offer extremely low thermal resistance, and have been studied as an emerging thermal interface material (TIM). In this work, we propose an improved form of LM/indium film/LM sandwich pad as an efficient TIM. The sandwich-like structure was designed to avoid liquid leakage and oxidation of LM, and additional micropillar arrays were fabricated on the surface, which benefitted the improved wetting of the substrate surface. A series of thermal tests revealed the anti-leakage characteristic and thermal stability of LM/indium film/LM, whose thermal resistance can also reach as low as 0.036 cm^2^ K W^−1^. Additionally, the heat dissipation test performed on a commercial smart phone demonstrated that a LM/In/LM pad not only reduced the temperature of the CPU and back cover but also enhanced the runtime of a battery by 25%

## Abstract 2

An improved form of LM/indium film/LM sandwich pad with surface micropillar arrays is a high-performance thermal interface material for thermal management.

## Graphical abstract

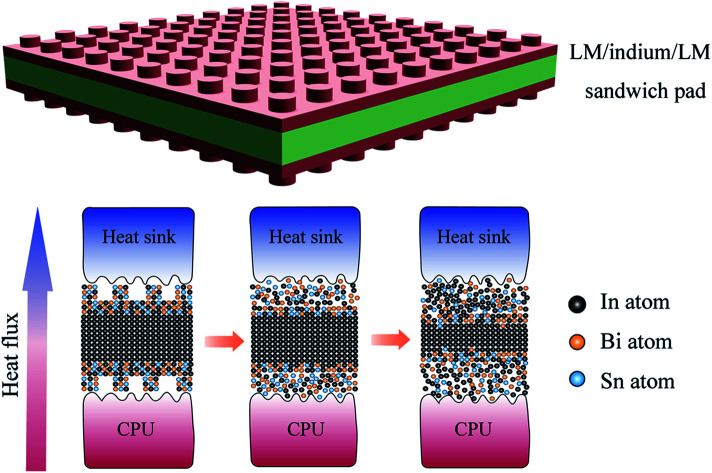

## Introduction

Given the continual increase in integration level and assembly density, electronic components generate a large amount of heat that cannot be dissipated timely and efficiently. This condition results in a notably high interface temperature in electronic components, which directly affects their life and stability.^1–5^ Therefore, the heat must be removed through effective thermal management to achieve a safe and stable working temperature. Effective thermal management depends on minimization of contact thermal resistance between interfaces between CPUs and heat sinks. When two solid surfaces are in contact, the maximum area between surfaces is filled with air possessing low thermal conductivity (0.026 W m^−1^ K^−1^ at room temperature).^6^ Therefore, the air gaps create a large thermal contact resistance that hinders heat conduction. Using thermal interface materials (TIMs) is an effective way to reduce thermal contact resistance to achieve fast and efficient heat transfer.^7,8^

Common TIMs include thermal grease, gel, thermal pad, phase change material, carbon-based TIM, and low-melting-point alloy.^9–14^ These TIMs are a mixture of polymers and thermal conductive particles, and their thermal conductivities are highly limited by polymers and interfaces between polymers and particles.^15^ In recent years, liquid metals (LMs), also called low-melting-point alloys, have been studied as an emerging TIMs owing to their superior properties, including high thermal conductivity, low thermal resistance, low viscosity, low toxicity, and low melting point temperatures typically below the running temperature of the CPU.^16–21^ LMs mainly include the following composites with different melting points: Ga_68.5_In_21.5_Sn_10_ (10 °C), Ga_90_In_10_ (15 °C), In_51_Bi_32.5_Sn_16.5_ (60 °C), In_66.3_Bi_33.7_ (72 °C), and In_26_Bi_57_Sn_17_ (79 °C).^22,23^ Martin and Kessel^24^ proposed a LM with an excellent thermal resistance of 0.02 cm^2^ K W^−1^ and effective thermal conductivity of 31 W m^−1^ K^−1^. Roy *et al.*^25^ reported that three low-melting alloys (Ga_75.5_In_24.5_, Ga_100_, and In_51_Bi_32.5_Sn_16.5_) survived for 3000 h of aging at 130 °C and 1500 cycles from −40 °C to 80 °C without significant thermal performance degradation. LMs can be used individually and also in combination with graphene or carbon nanotubes for TIM applications.^26–28^ Although LMs possess superior thermal properties, they feature several issues such as oxidation, intermetallic growth, dewetting and leakage.^29^ Among these issues, the most severe problem is the leakage of an LM, because it will short the circuit and cause instant failure of the device. Although a plastic or paste sealing around the LM may solve the problem,^30^ the sealing will compromise the thermal resistance and result in low reliability.

**Fig. 1:**
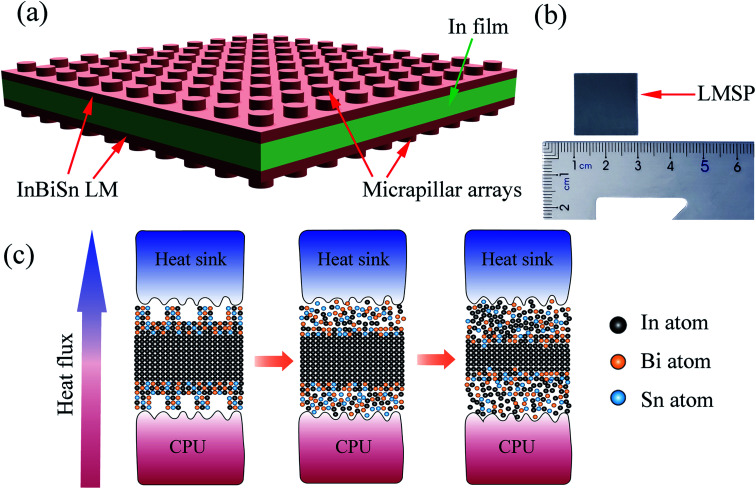
(a) Schematic of LMSP TIM; (b) photograph of a LMSP with the size of 20 mm × 20 mm; (c) melting-absorption microscopic mechanism of LMSP—the left image shows the LMSP placed between the CPU and the heat sink; the middle image presents the micropillars that deform and fill the gap under pressure; the right image displays the LM melting when heated and then absorbed by the indium film to form a solid again.

In this work, we propose an improved form of a LM film with a sandwich structure: LM/indium film/LM with surface micropillar arrays [Fig. 1(a)]. The indium film was used as the center layer because indium is widely used as TIM owing to its extreme softness with the hardness of 1.2 HB. However, indium is notably hard to use when wetting the thermal interface. As a result, we considered extra InSnBi LM layers on the top and bottom of the indium foil to facilitate the low interface thermal resistance. In such a sandwich structure, when the CPU temperature rises above the melting point of LM, the LM expectedly melts and wets the CPU's and heatsink's surface. Meanwhile, given that InSnBi is chemically similar to indium, additional molten InSnBi will be absorbed and incorporated into the indium film, as shown in Fig. 1(b). As a result, no LM will be leaked. Additionally, during the absorption process, the InSnBi/indium interface becomes chemically bonded, which will minimize the interfacial resistance between them. Moreover, herein, micropillar arrays were imprinted on the surface of LM/In/LM pad to fill considerably smaller interface gaps, improving the wettability of LM and further reducing the interface thermal resistance. In the experiment, the sample [Fig. 1(c)] demonstrated a low thermal resistance of 0.036 cm^2^ K W^−1^ with superior anti-leaking capability. LM SANDWICH PAD also revealed a durable performance on thermal tests, reduced the CPU temperature by ∼5 °C, and enhanced the runtime of battery by as much as 25%.

### Experiment

InBiSn LM with 60 °C melting point was prepared from high-purity indium, bismuth, and tin (In : Bi : Sn = 51 : 32.5 : 16.5 by weight, Wuhan Xinrong New Materials Co., Ltd). These raw materials were mixed into a ceramic crucible, heated to 800 °C, and kept for 4 h in a high-temperature vacuum furnace (GWL-1600ZKLB, Luoyang Guoju Furnace Co., Ltd). The acquired InBiSn LM was rolled into sheets through a calendaring machine (MSK-2150, Shenzhen Kejingstart Technology Co., Ltd). Then, an indium film was used as the center layer between two LM sheets through rolling, and a sandwich-like material was obtained. Finally, the micropillar arrays were imprinted on the surfaces of the LM SANDWICH PAD through a thermal nanoimprint device (PP-HENIL-02, Nanjing Pengpai New Material Technology Co., Ltd). Here, the nanoimprinting method used a silicon template that would not adhere to molten InBiSn LM. Thus, micropillar arrays structure can be transferred from the silicon template to LM/In/LM pad through direct contact at certain temperature and pressure.

A field emission SEM was used to investigate surface morphological and cross-section of the sample, whereas spatial distribution of elements was analysed by EDS. Thermal resistance was estimated by an ASTM D5470 standard TIM tester (DRL-III, Xiangtan Xiangyi Instrument Co., Ltd). A series of essential thermal tests, including accelerated aging test and thermal cycling test, was carried out by placing LM/In/LM pad, which was sandwiched by two copper sheets into a programmable temperature and humidity chamber (LY280, Dongguan Liyi Experiment Instrument Co., Ltd). A commercial silicon pad (Laird T-flex 600) and the LM/In/LM pad were installed in the CPU of a smartphone (Y67a, Vivo). Then, the smartphone was placed in the programmable temperature and humidity chamber to maintain a constant atmosphere throughout the test period. Stability Test v2.7 software was used to run the CPU with 100% load, and the temperature increase of the smartphone was measured by a multiplex temperature tester (Jinko, JK-8U). Infrared thermal images of the same phone were obtained by an FLIR E4 camera.

## Results and discussion

**Fig. 2:**
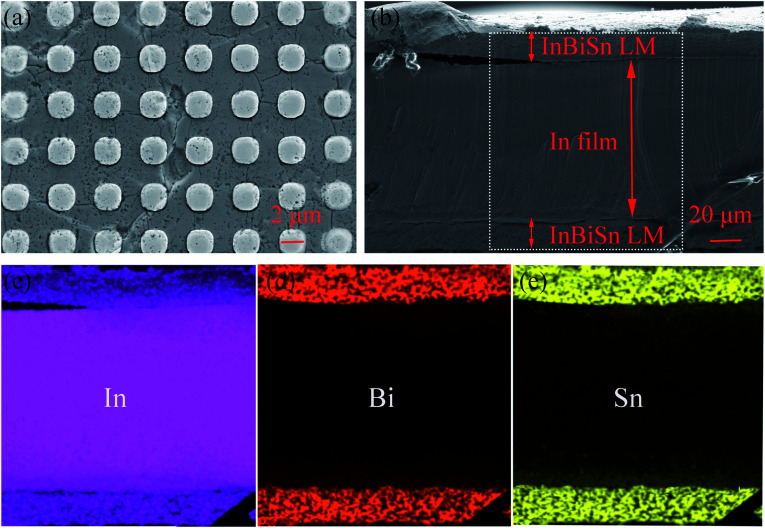
(a) SEM image of micropillar arrays on the LMSP surface; (b) SEM cross-section image of LMSP demonstrating the sandwich-like structure. (c), (d), and (e) EDS elemental mapping of the cross-section presenting the spatial distribution of In, Bi, and Sn, respectively.

Fig. 2(a) shows the SEM image of micropillar arrays, whose diameter and pitch, both are about 2 μm. Fig. 2(b) shows the cross-section image of the sandwich-like structure. The thicknesses of the three layers were about 20, 100, and 20 μm. EDS elemental mapping was performed on the whole cross-section of the LM/In/LM pad to identify the spatial distribution of different elements, *i.e.*, In, Bi, and Sn, as shown in Fig. 2(c), (d), and (e), respectively. The interlayer mostly included In, whereas two surface layers contained In, Bi, and Sn, which further verified the sandwich-like structure of LM/In/LM.

**Fig. 3:**
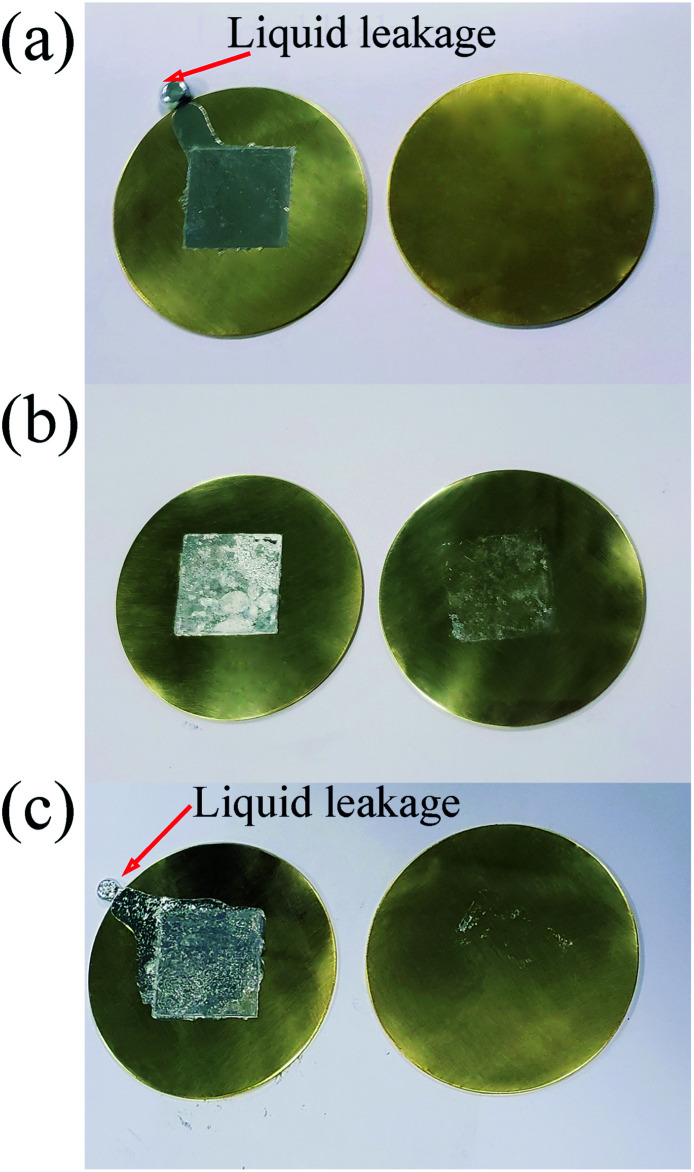
(a) Liquid leakage of 0.1 mm-thick InBiSn LM foil between copper sheets after melting and cooling; (b) LMSP sample with 20 μm-thick LM between copper sheets after melting and cooling; (c) liquid leakage of sample with 50 μm-thick LM between copper sheets after melting and cooling.

Liquid leakage is a great risk when LMs are used as TIM. Although InBiSn LM was a thin foil with 0.1 mm thickness, the excess materials were extruded out from the copper sheets after melting as shown in Fig. 3(a). However, when we used two 20 μm-thick LM foils and a 200 μm thick LM indium film to form a sandwich-like pad as TIM, no liquid leakage occurred after melting as shown in Fig. 3(b). This concise comparison effectively proved the anti-leakage property of LM/In/LM pad. However, when LM thickness was increased to 50 μm, the molten LM exceeded the amount that indium can absorb, and liquid leakage occurred as shown in Fig. 3(c).

**Fig. 4:**
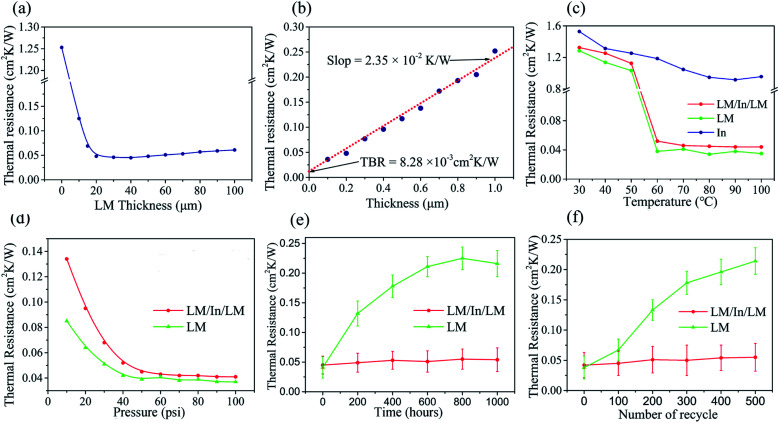
(a) Thermal resistance of LM/In/LM with different InBiSn LM thicknesses; (b) thermal resistance of LM/In/LM with different thicknesses of indium film; (c) thermal resistance *vs.* temperature curve showing the temperature effect on the thermal resistance of LM/In/LM; (d) thermal resistance *vs.* pressure curve showing the pressure effect on the thermal resistance of LM/In/LM; (e) thermal resistance *vs.* aging time showing the changes in thermal resistance after aging at different times; (f) thermal resistance *vs.* cycle curves showing the changes in thermal resistance at different thermal cycling numbers.

The thermal resistance of pure indium (200 μm-thick) and LM/In/LM pads with the same indium and LMs with different thickness was measured at 80 °C and 50 psi, as shown in Fig. 4(a). The pure indium without LM had a high thermal resistance of 1.253 cm^2^ K W^−1^ given the difficulty of using solid indium to wet the thermal interface. Given that LM pieces were used to sandwich the indium film, the thermal resistance of samples dropped notably. When LM thickness was increased from 10 to20 μm, the thermal resistance decreased from 0.125 cm^2^ K W^−1^ to 0.048 cm^2^ K W^−1^, which indicated that 20 μm-thick LM layer could wet the thermal interface more efficiently. When LM thickness was continually increased from 20 to 40 μm, there was no significant improvement to the thermal resistance. And it increased slowly from 0.048 cm^2^ K W^−1^ to 0.061 cm^2^ K W^−1^ when keeping increasing the thickness from 50 to 100 μm. Therefore, the best thickness of the LM layer is 20 μm, which could reduce thermal resistance efficiently and avoid leakage from excessive LM.

Thermal conductivity is an important parameter for TIM. The thermal resistance *versus* indium thickness curve [Fig. 4(b)] at 70 °C and 50 psi was constructed to calculate the thermal conductivity. The data demonstrated a linear relationship that can be expressed as follows:*R* = 2.35*t* + 8.28 × 10^−3^ cm^2^ K W^−1^where *R* is the thermal resistance; *t* is the thickness of bulk materials. Thus, the thermal conductivity, as the inverse of the slope, was 42.6 W m^−1^ K^−1^. This value was larger than the thermal conductivities of most commercial TIMs and those reported in literature. Meanwhile, the thermal contact resistance was as low as 8.28 × 10^−3^ cm^2^ K W^−1^, which is due to the excellent wettability of the LM and copper substrate.

The effect of interface temperature on the thermal resistance of In film (100 μm-thick), single LM sheet (20 μm-thick) and LM/In/LM pad (140 μm-thick) were observed in Fig. 4(c). These samples were measured from 30 °C to100 °C with the pressure of 50 psi. The thermal resistance of LM and LM/In/LM was extremely high under 60 °C and dropped rapidly once the interface temperature reached 60 °C. A phase change occurred on the InBiSn alloy, and LMs filled the gaps between the interfaces effectively, reducing the thermal resistance of LM (0.038 cm^2^ K W^−1^) and LM/In/LM (0.052 cm^2^ K W^−1^). No phase change occurred to In, so its interface contact can't be improved, with the thermal resistance above 1 cm^2^ K W^−1^. The study also concluded that when LMs melt and bond with the interface, their thermal resistance can be hardly affected by interface temperature. Besides, compared to single LM sheet, the thermal resistance of LM/In/LM pad that including two LM sheets does not increase significantly, indicating that the design of the sandwich structure is feasible.

Fig. 4(d) presents the thermal resistance of LM/In/LM sandwich pad as function of applied pressure, where the interface temperature was applied at 70 °C. The thermal resistance of LM sheet decreased from 0.085 cm^2^ K W^−1^ to 0.037 cm^2^ K W^−1^ with the pressure from 10 to 100 psi, while the thermal resistance of LM/In/LM changed from 0.134 cm^2^ K W^−1^ to 0.041 cm^2^ K W^−1^. We conclude that appropriate pressure can improve the quality of interface contact and enlarge the real contact area. When the applied pressure reached a certain value, the melting alloys would be forced into most of the interstitial voids, and minimal change was observed with the thermal resistance. Although the pressure applied on the LM sheet is smaller than LM/In/LM, it is easier to come out from the interface, which cause leaking.

Reliability tests, including aging test and thermal recycling test, were conducted for the LM/In/LM pad. The accelerated aging test was carried out by exposing the Cu disks assembly with LM sheet and LM/In/LM pad at 85 °C and 85% relative humidity for extended periods. Fig. 4(e) shows the effect of aging time on thermal resistance, where samples were measured at 70 °C and 50 psi. As for LM sheet, the thermal resistance increased from 0.041 cm^2^ K W^−1^ to 0.216 cm^2^ K W^−1^ after 1000 h of aging. LM sheet melted all over 60 °C and both vapor and oxygen are easy to diffuse into molten alloys, which cause severe oxidation degradation. LM/In/LM pad can survive for as long as 1000 h of aging without significant performance degradation. It is because indium can react with oxygen and formed an indium oxide layer as protection, which effectively prevent vapor and oxygen. Additionally, the superior aging performance was attributed to the favorable wetting between the LM and copper disk, because indium atoms from LM easily diffused and formed an intermetallic portion with the Cu surface. The In–Cu interaction resulted in efficient and stable interface contact; thus, LM/In/LM could maintain low thermal resistance.^24^

Thermal cycling tests were carried out to cycle LM sheet and LM/In/LM pad from −40 °C to 120 °C. All samples were placed in a thermal cycling chamber, where heating and cooling were performed at a ramp rate of 11 °C min^−1^ and then kept for 30 min at the two extreme temperatures. The thermal resistance of LM sheet and LM/In/LM pad were measured at 70 °C and 50 psi after different numbers of thermal cycling as shown in Fig. 4(f). A total of 23.8% degradation was observed for the thermal resistance, which increased from 0.042 cm^2^ K W^−1^ to 0.052 cm^2^ K W^−1^. This phenomenon occurred mainly because micropillar arrays promoted the quality of thermal contact and enhanced wetting of the interface. The interface reaction between the InBiSn alloy and Cu substrate also formed a durable thermal contact, which was hardly affected by heating and cooling.^29^ However, obvious degradation occurs to LM sheet with thermal resistance increasing from 0.038 cm^2^ K W^−1^ to 0.214 cm^2^ K W^−1^. During the thermal cycling tests, expansion and contraction occurred to LM with temperature rising and dropping because of the migration and dewetting of LM. The excess molten alloys extruded out of the interface and voids were created, increasing the thermal resistance.

**Fig. 5:**
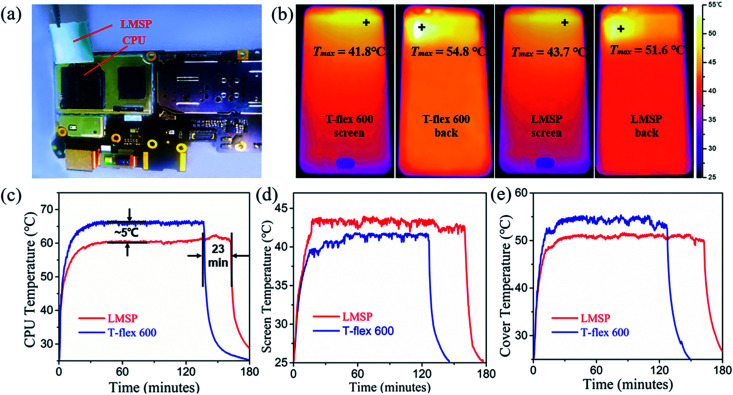
(a) Photo of the LMSP used as TIM on the smartphone; (b) infrared thermal images of the back cover and front cover of the same smartphone with LMSP and T-flex 600 thermal silicon pad; (c) curves of CPU temperature increase measured with commercial T-flex 600; (d) increase in front-screen temperature of LMSP and T-flex 600; (e) increase in back-cover temperature of LMSP and T-flex 600.

The LM sandwich pad (12 mm × 14 mm × 20 μm) was applied in a smartphone (Y67a, Vivo), as shown in Fig. 5(a), to further verify its applicability in devices. LM/In/LM pad was placed in a chamber with a constant temperature of 25 °C and 60% relative humidity; the smartphone was used to run Stability Test v2.7 software to operate the CPU with high load. As shown in Fig. 5(c), the CPU temperature of the smartphone equipped with LM sandwich pad increased to ∼62 °C and lasted for 160 min until the battery ran out. Under the same thermal shock, a thermal silicon pad Laird T-flex 600 was applied to the CPU. The CPU with T-flex 600 showed a larger temperature rise than LM/In/LM pad, and the temperature drop reached ∼5 °C. Moreover, the smartphone using T-flex 600 ran 23 min less than that using LM sandwich pad. When heat generation and dissipation reach a balance, the temperature of CPU will not rise anymore. A low temperature means a low heating power for the CPU with the largest frequency. Therefore, the CPU using LM/In/LM pad can save more energy, and the battery can last for a long time. This finding indicates the excellent heat dissipation performance LM/In/LM pad because of its smaller thermal resistance. Meanwhile, the back-cover temperature of the smartphone using LM/In/LM pad was lowered down by 3.2 °C [Fig. 5(d)], whereas the front-screen temperature rose by ∼2 °C compared with T-flex 600 [Fig. 5(e)]. Fig. 5(b) presents the infrared thermal images of smartphone applying LM/In/LM pad and T-flex 600; the images confirmed the temperature change in the front screen and back cover. The overall heat generated by CPU remained the same, but the amount of heat transferred to the front screen *via* LM/In/LM pad increased, whereas the amount of heat transferred to the back cover decreased. The temperature rise on the back cover can improve the user experience because users are more likely to touch this part and are insensitive to the heat from the screen.

## Conclusion

In this study, we proposed an improved form of LM/indium film/LM sandwich with micropillar arrays pad as TIM to meet practical applications for electronic components. The unique sandwich structure was designed to avoid liquid leakage while maintaining good thermal resistance. SEM image and EDS mapping confirmed the sandwich structure. High thermal conductivity of 44 W m^−1^ K^−1^ was calculated from thickness *versus* thermal resistance curve; this value is higher than that of most TIMs that have been reported. The effect of contact pressure and interface temperature on thermal tests showed that LM/In/LM pad offers low thermal resistance as low as 0.036 cm^2^ K W^−1^ above 60 °C at 50 psi. The LM/In/LM pad also revealed a durable thermal performance without severe degradation after 200 h of accelerated aging and 200 cycles from −40 °C to 120 °C, respectively. The heat dissipation performance test proved the strong temperature drop of 5 °C at the CPU and 4 °C at the back cover of LM/In/LM pad compared with T-flex 600 thermal silicon pad. More importantly, LM/In/LM pad enhanced the runtime of battery by as much 25%, indicating that LM/In/LM pad is an energy-efficient and commercially available TIM for thermal management.

## Conflicts of interest

There are no conflicts to declare.

## Supplementary Material
